# Growing Inequities in Median Age of Death and Obesity Prevalence Between Australian Major Cities and Remote Areas: A National, Longitudinal, Spatial Analysis

**DOI:** 10.1002/puh2.70189

**Published:** 2026-01-29

**Authors:** Jonathan R Olsen, Thomas Astell‐Burt, Xiaoqi Feng

**Affiliations:** ^1^ Institute for Social Science Research The University of Queensland Australia; ^2^ School of Architecture, Design and Planning University of Sydney Sydney Australia; ^3^ Population Wellbeing and Environment Research Lab (PowerLab) Sydney Australia; ^4^ Westmead Applied Research Centre, Westmead Hospital Sydney Australia; ^5^ Charles Perkins Centre University of Sydney Sydney Australia; ^6^ Sydney Environment Institute University of Sydney Sydney Australia; ^7^ School of Population Health University of New South Wales (UNSW) Sydney Australia; ^8^ The George Institute for Global Health Sydney Australia

## Abstract

**Background:**

Obesity significantly contributes to longevity, with 25‐year‐old Australians experiencing reduced life expectancy of 8.3 years (men) and 6.1 years (women) compared to healthy‐weight peers. Given Australia's diverse geographical composition, this study examined trends in median age of death and obesity prevalence by remoteness over time.

**Methods:**

Median age of death and modelled obesity prevalence data were obtained from the Public Health Information Development Unit (PHIDU) Social Health Atlas at Population Health Area level for six time points (2010–2022) and four time points (2011–2022), respectively. Data were linked to state (*n* = 8), Remoteness Area (*n* = 5) and Socio‐Economic Indexes for Areas (SEIFA) deciles. Mixed‐effect linear regression models examined differences by remoteness while adjusting for state/territory and area‐level deprivation. Temporal inequities were assessed using the Slope Index of Inequality (SII).

**Results:**

Persistent and widening inequities in median age of death (2018–2022: male SII −3.6, 95% CI −4.9 to −2.3; female SII −2.5, 95% CI −3.8 to −1.2) and obesity (2022: male SII 11.7, 95% CI 10.4–12.9; female SII 12.5, 95% CI 11.1–13.9) were observed between major cities and remote areas. Obesity prevalence increased across all Australian areas, with smallest increases in major cities (men 3, women 0.5 percentage points) but substantially greater increases in outer regional areas (men 11, women 7 percentage points), creating a clear divergence in geographic inequity. Marked disparities in obesity prevalence existed across major cities, regional and remote areas.

**Conclusions:**

Geographic disparities were apparent in obesity prevalence and median age of death, as well as increasing inequities in both median age of death and obesity prevalence among Australian major cities, regional and remote areas. These findings highlight the urgent need for targeted public health interventions, particularly in regional and remote areas, to address Australia's growing health inequities.

## Graphical abstract

Geographic health inequities in median age of death and obesity prevalence widened across Australia between 2010 and 2022. Individuals living in remote areas of Australia exhibited lower median age of death and greater obesity prevalence compared to those residing in major cities.

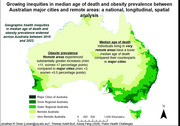

## 1 Introduction

Life expectancy varies globally, with marked differences across regions, levels of socio‐economic development and both within‐and‐between countries [1]. Australian life expectancy ranks among the highest in the world, holding fourth place among the 38 member nations of the Organisation for Economic Co‐operation and Development (OECD) [2]. Nationally, life expectancy at birth is 85 years for women and 81 years for men [3]. Despite its relatively high ranking, Australia demonstrates one of the widest gaps between life expectancy and health‐adjusted life expectancy (HALE) among World Health Organization (WHO) member states—a measure often referred to as the ‘healthspan‐lifespan gap’ [4]. Around half of Australians report living with at least one chronic condition [5], with one‐quarter experiencing a mental or behavioural disorder. Non‐communicable diseases linked to modifiable risk factors—such as tobacco use, overweight and obesity, unhealthy diet, physical inactivity, hypertension and alcohol use—make substantial contributions to the overall burden of disease [6, 7].

Overweight and obesity are particularly important contributors to premature mortality [8]. Obesity in mid‐life has been associated with increased risk of a number of adverse conditions, such as cardiovascular disease, Type 2 diabetes and certain cancers [9]. Estimates suggest that an obese 25‐year‐old Australian may experience a reduction in life expectancy of 8.3 years for men and 6.1 years for women compared with peers of healthy weight [10]. However, the broader impact of obesity on population‐level life expectancy may be underestimated; rather than causing sharp declines, obesity likely slows or halts further gains in life expectancy by disproportionately affecting healthy life expectancy [11]. However, with the projected rising levels of obesity in Australia [12], the impact on life expectancy may become more pronounced over time.

Many studies do not capture inequities in health outcomes both between and within regions as well as rural and remote areas, and their trajectories over time [13]. In Australia, life expectancy varies across states for both men and women, with the Northern Territory consistently recording lower levels than other states [14, 15]. The Northern Territory is geographically vast and includes a major urban centres (e.g., Darwin, estimated population: 87,608 [proportion of total population: 34.3%] alongside many rural and remote settlements [16], with diverse population demographics [17]. In addition to geographical inequalities, differences in life expectancy have been observed by socio‐economic status within Australia, which are inextricably linked [11], and these inequities have increased over the past decade [18]. For obesity, area‐level socio‐economic status was found to be a predictor of BMI for both men and women [19]. In addition, longitudinal analysis and future simulations highlight persistent and widening inequalities in obesity for Australian adults across socio‐economic groups over time [12, 20]. These intersecting disparities, combined with distinct population profiles across levels of remoteness, underscore the importance of examining how trends in longevity and obesity differ by geographic context, whist controlling for key socio‐economic variables. In addition, researchers examining rural health disparities have highlighted that socio‐economic variables should be included in analysis of mortality outcomes as many disparities are related to income and poverty [21]. This study extends existing literature and addresses a critical gap in the literature by simultaneously examining temporal trends in median age of death and obesity across Australia's remoteness areas while formally quantifying inequities, whilst adjusting for socio‐economic confounding. Our longitudinal approach reveals not only persistent disparities but accelerating divergence between major cities and remote areas, providing crucial evidence for geographically targeted public health interventions.

This study aims to: (1) investigate temporal trends in median age of death and obesity across different levels of remoteness in Australia; (2) assess whether distinct regional patterns have emerged; and (3) evaluate the extent of inequities between these areas.

## 2 Methods

### 2.1 Data

Median age of death and obesity data were obtained from the Public Health Information Development Unit (PHIDU) Social Health Atlas (https://phidu.torrens.edu.au/) from 2021 to 2022 (earliest and most recent data), which provides health and social statistics for small areas in Australia. Data extraction periods corresponded to those published by PHIDU, with the earliest and most recent releases for small‐area units included in this study.

All data were downloaded at the Population Health Area (PHA) level. PHAs are small‐area statistical units derived from the Australian Bureau of Statistics (ABS) Statistical Areas Level 2 (SA2) using the Australian Statistical Geographical Standard (ASGS). They were specifically created to enable dissemination of health and social statistics in instances where SA2‐level data contained too few cases for reliable analysis or public release. In Australia there are 1165 PHA's.

#### 2.1.1 Median Age of Death

Median age at death was extracted at the PHA level for men and women at six time‐points: 2010–2014, 2013–2017, 2015–2019, 2016–2020, 2017–2021 and 2018–2022. The aggregated statistic over 5‐year period is used to smooth out any individual year fluctuations and improve the reliability of estimates for small area calculations. These data were calculated by PHIDU using Cause of Death Unit Record Files supplied by the Australian Coordinating Registry and the Victorian Department of Justice [22].

#### 2.1.2 Obesity Prevalence

Modelled, age‐standardised obesity prevalence estimates (per 100 population) were extracted at the PHA level for men and women aged ≥ 18 years for the periods 2011–2012, 2014–2015, 2017–2018 and 2022. Obesity was defined within the dataset as a body mass index (BMI) ≥ 30. Estimates were modelled based on responses from approximately 60% of individuals aged ≥ 18 years who participated in the ABS National Health Survey and had their height and weight measured objectively [22].

#### 2.1.3 Area‐Level Socio‐Economic Advantage and Disadvantage

Socio‐Economic Indexes for Areas (SEIFA) 2021 deciles for each PHA were downloaded from PHIDU, which were constructed using population weighted averages based on the published ABS SA2 data [23]. SEIFA uses a number of census variables (income, education, employment, occupation, housing and family structure) that provide indicators of advantage and disadvantage for small areas.

### 2.2 Spatial Data Linkage

Median age of death and obesity prevalence data were downloaded for all Australian PHAs for each individual time point. PHAs were spatially linked and classified according to the federal state (*n* = 8) and Remoteness Area (*n* = 5) categories in which they were located. The Australian Statistical Geography Standard (ASGS) Remoteness Area classification divides Australia into five categories: Major Cities, Inner Regional, Outer Regional, Remote and Very Remote. This classification is based on road distance to the nearest urban centre or locality, providing an indicator of access to services [24].

Where PHA and remoteness area boundaries did not align perfectly, remoteness was assigned using the ‘Join Attributes by Location’ tool in QGIS Desktop (version 3.20.0). In cases of partial overlap, the remoteness category was allocated based on the area of greatest spatial overlap (one‐to‐one assignment). Figure S1 shows the remoteness categories with PHA boundaries overlaid for Australia.

### 2.3 Analysis

Median age of death and obesity prevalence were reported for Australia overall and stratified by remoteness for each time‐point. Differences in outcomes between remoteness categories were assessed using analysis of variance (ANOVA), with *p*‐values reported to indicate statistically significant differences. This provided an initial descriptive investigation of associations between remoteness categories and outcomes before formal testing with covariate adjustment in subsequent models.

### 2.4 Change Over Time in Median Age of Death and Obesity by Remoteness

To further examine differences by remoteness while adjusting for state and territory as a fixed‐effect and area‐level deprivation (SEIFA deprivation decile), we fitted mixed‐effect linear regression models of median age of death including remoteness, year and their interaction. PHA was specified as the clustering variable to account for clustering of observations within geographic areas (which does not fully account for spatial autocorrelation between neighbouring areas) and random intercept to account for the repeated measures overtime at this spatial unit. Adjusted marginal means were derived and plotted to visualise temporal trends. For age‐standardised obesity prevalence, which was available only at the area level without denominators, a mixed‐effect linear regression model was also applied. The obesity prevalence data were normally skewed across the PHA areas, suggesting this approach was appropriate.

### 2.5 Differences in Median Age of Death and Obesity Across Remoteness Categories Over Time, Using a Slope Index of Inequality

To assess the magnitude of differences in outcomes by remoteness over time, inequities across the remoteness gradient were summarised using the Slope Index of Inequality (SII) [25, 26]. We calculated population‐weighted ridit scores for each remoteness category by assigning each small area a score representing the midpoint of its cumulative population distribution. These ridit scores provided a continuous remoteness rank that was then used in mixed‐effect regression models to estimate the SII, ensuring that all groups and their population sizes were incorporated into the inequity measures [27]. Median age of death and obesity prevalence were then regressed on the ridit score, period and state, including an interaction between ridit and year. Marginal estimates were used to obtain period‐specific SIIs, enabling assessment of changes in inequity over time, by gender.

All analyses were performed within StataMP 18. The formulas for the two main models and code used to fit each model have been included in the Supporting Information.

## 3 Results

### 3.1 Trends in Median Age of Death and Obesity Prevalence in Australia

Median age of death in Australia has shown minimal change between 2010–2014 and 2018–2022, increasing by approximately 1 year for men and 0.5 years for women (Table 1). Women continue to have a higher median age of death than men, with a 5.5‐year gap observed in 2018–2022. Marked differences are also evident by geographic remoteness, with residents of major cities living on average, a decade longer than those in very remote areas.

**TABLE 1:** Median age of death outcomes overtime by remoteness, male and female.

	Men						Women					
Remoteness areas	2010–2014	2013–2017	2015–2019	2016–2020	2017–2021	2018–2022	2010–2014	2013–2017	2015–2019	2016–2020	2017–2021	2018–2022
Major cities	78.0	78.0	78.0	79.0	79.0	79.0	83.5	84.0	84.0	84.0	84.0	84.0
Inner regional	77.0	78.0	78.0	77.5	78.0	78.0	83.0	84.0	83.0	83.0	83.0	83.0
Outer regional	76.8	77.0	77.0	77.0	77.0	77.0	82.0	83.0	82.0	82.0	82.3	83.0
Remote	77.0	77.0	77.0	75.0	75.3	76.0	84.0	83.8	83.5	81.5	82.0	82.0
Very remote	69.3	70.5	71.5	68.5	68.5	69.0	74.0	73.0	74.0	67.0	68.5	69.8
**Australia**	77.0	78.0	78.0	78.0	78.0	78.0	83.0	83.5	83.0	83.0	83.0	83.5
*F*	29.8	29.2	28.2	61.2	58.7	40.5	30.1	27.5	26.5	56.4	57.1	62.2
*p*	< 0.001	< 0.001	< 0.001	< 0.001	< 0.001	< 0.001	< 0.001	< 0.001	< 0.001	< 0.001	< 0.001	< 0.001

The prevalence of obesity in Australia increased from 28% in 2011–2012 to 34% in 2022, representing a six‐percentage‐point rise over the past decade (Table 2). Across all survey years, obesity prevalence was consistently lower among residents of major cities compared with those living in regional and remote areas.

**TABLE 2:** Obesity prevalence over time by remoteness, men and women.

Remoteness areas	Men				Women			
	2011–2012	2014–2015	2017–2018	2022	2011–2012	2014–2015	2017–2018	2022
Major cities	28.3	27.8	30.6	30.1	29.1	27.1	28.5	28.3
Inner regional	27.7	33.0	37.1	38.0	29.8	31.8	34.5	36.2
Outer regional	29.2	33.6	38.4	39.1	30.9	31.3	35.8	37.2
Remote	31.1	32.2	37.2	36.0	29.7	31.4	34.1	34.3
Very remote	30.9	30.3	40.4	37.2	28.5	30.4	37.4	35.6
**Australia**	28.3	30.2	33.8	34.0	29.4	29.3	31.7	32.2
*F*	6.71	34.3	79.87	86.46	2.42	26.64	74.02	90.57
*p*	< 0.001	< 0.001	< 0.001	< 0.001	< 0.001	< 0.001	< 0.001	< 0.001

### 3.2 Differences in Median Age of Death and Obesity Prevalence by Remoteness Area Classification

**FIGURE 1:**
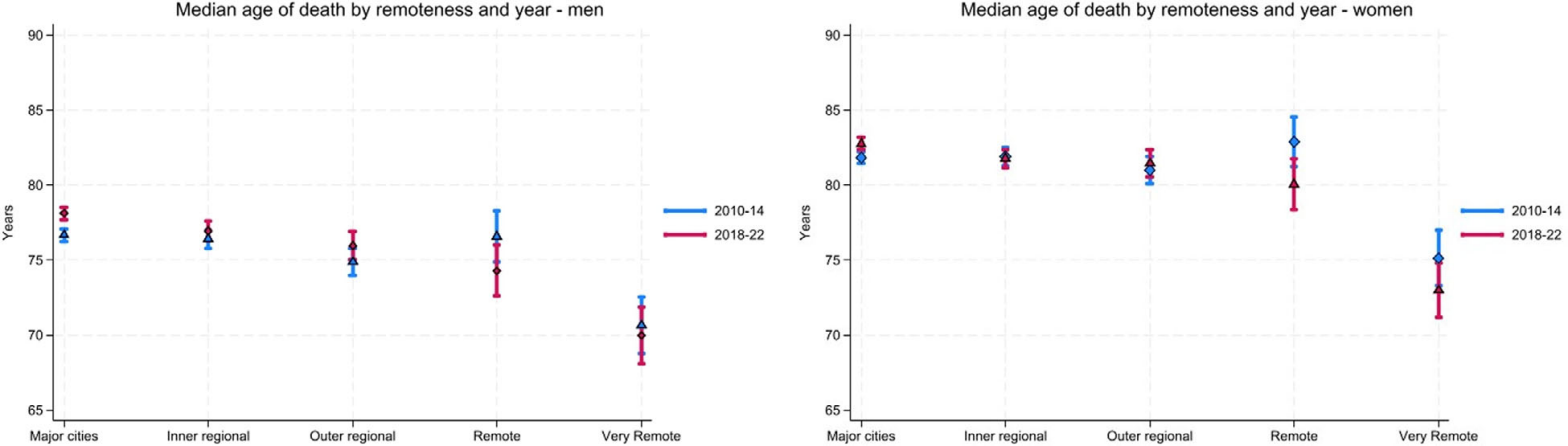
Model‐adjusted median age of death by remoteness and year, men and women, 2010–2014 to 2018–2022. *Note:* Absolute values shown in Tables S1 and S2.

**FIGURE 2:**
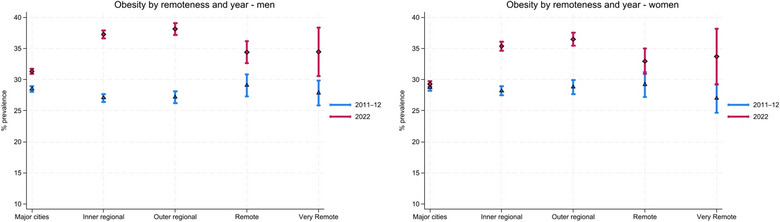
Model‐adjusted obesity by remoteness and year, men and women, men and women, 2011–2022. *Note:* Absolute values shown in Tables S3 and S4.

Figure 1 presents median age of death over time by remoteness area classification for the first (2010–2014) and last (2018–2022) periods of observation, adjusted for state (two time points are displayed for clarity; full model outputs are provided in Tables in Supporting Information). Overall, median age of death was consistently lower among individuals residing in very remote areas compared with those in major cities, and this disparity persisted across time. For men residing within major cities, there was a significant increase in median age of death of 0.9 years from 2010–2014 to 2018–2022. No other substantive changes in median age of death were observed within individual remoteness categories across the study period (Figure 2).

Obesity prevalence increased across all regions of Australia for both men and women Figure 2). The smallest increases were observed among residents of major cities (men: +3 percentage points; women: +1 percentage point), whereas the largest increases occurred in outer regional areas (men: +11 percentage points; women: +8 percentage points).

### 3.3 Inequity in Median Age of Death and Obesity Prevalence by Remoteness Area Classification

**FIGURE 3:**
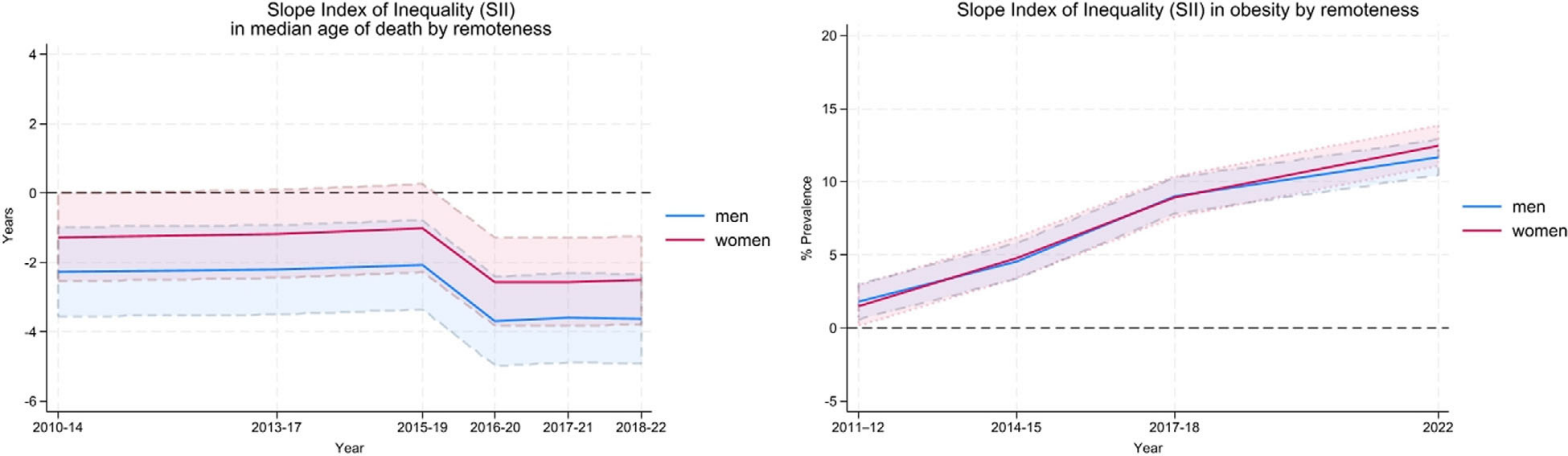
Slope Index of Inequality (SII) in median age of death and obesity by remoteness, men and women, 2010–2022. *Note:* Absolute values shown in Table S5.

Figure 3 presents the absolute gap in median age of death (years) and obesity prevalence between the least and most remote areas of Australia, estimated using the SII across all five remoteness categories.

For median age of death, the SII increased from −1.3 years in 2010 to −2.5 years in 2018 for women and −2.3 to −3.6 years for men, with a marked shift in trend from 2015 to 2019, a period which includes the COVID‐19 pandemic, highlighting widened rural–urban inequities. No significant gender differences were observed in the SII for median age of death.

For obesity, the SII has risen steadily from 2011 to 2012, where there was an absolute difference of between 1.5 and 1.8 percentage points in obesity between the very remote areas and major cities, to a 12‐percentage point difference in 2022 for men and women. The most recent time‐period highlighting significant inequities between very remote residents compared to those in major cities. Again, no substantive gender differences were evident in the SII for obesity.

## 4 Discussion

### 4.1 Summary of Results

Our findings demonstrate persistent and widening inequities in both median age of death and obesity between Australians living in major cities and those in remote areas. Median age of death differences by remoteness classification remained stable over time with the exception of a one‐time persistent increase. No other substantive improvements or declines observed. However, overall inequities by remoteness worsened over time, with a pronounced shift between 2015–2019 and 2016–2020, when the SII increased by approximately 1 year.

For obesity, marked disparities in prevalence were observed across remoteness categories. Obesity prevalence increased across all areas of Australia, these were smallest in major cities but substantially greater in outer regional areas, resulting in a clear divergence in inequity.

### 4.2 Comparison With Other Literature

Globally, life expectancy has followed two major temporal trends since the 1950s. First, it rose steadily for several decades, and in some areas this trajectory began to level, until the COVID‐19 pandemic in 2020 led to a reversal and modest declines in many countries [28]. Even before the COVID‐19 pandemic, projections suggested that increases in Australian life expectancy between 2015 and 2025 would slow and could even plateau or decline slightly [29]. From 2020 onwards, consistent with global patterns [30], Australia experienced a modest reduction in life expectancy, though the extent of decline varies across states [31]. Our results highlight that as well as stalling trends in median age of death in Australia, inequities between remote areas and major cities are increasing. When combined with evidence of widening socio‐economic inequities [18], this highlights significant concern for a growing gap between population groups living in certain areas, their socio‐economic position and subsequent health outcomes. Evidence from other regions, such as the United States shows similar trends in urban–rural disparities, which are growing [32].

Rural populations across many global contexts exhibit lower life expectancy and poorer health outcomes compared to urban residents, as documented in the United States [33, 34], Canada [35] and the United Kingdom [36]. These health disparities are attributed to multiple interconnected factors including reduced healthcare access [37], lower quality educational opportunities [38], higher poverty rates [32], limited health literacy [39] and environmental context, which can impact on negative exposures and foster adverse health risk behaviours [40]. In rural Australia specifically, these disparities are further compounded by persistent youth out‐migration, whereby healthier and more educated young people relocating out of remote and regional areas to major cities, leaving behind increasingly vulnerable populations [41]. These factors are likely contributing factors in the growing inequities we show in our findings between major cities and remote areas for longevity and obesity outcomes. In addition, Australia has experienced substantial internal migration across cities and states; between 2020 and 2021 annual estimates show that 370,549 Australians moved interstate [42]. Internal migration data aren't often reported or reflected in administrative databases in a timely manner, which may impact on the population denominator data or where deaths occur and are registered.

Our findings reveal a clear geographic gradient in obesity prevalence, with rates lowest in inner city areas and highest in very remote regions. This relationship for Australian adults has been documented in official statistics, as well as the larger rise for a combined regional and remote area classification compared to major cities [43]; in our analysis we highlight marked differences when exploring trends by remote, inner and outer regional area classifications.

Notably, the greatest temporal obesity prevalence increases occurred in inner and outer regional areas rather than the most remote locations. This pattern reflects the complex relationship between geographic location and obesogenic environments. Urban residents, particularly those in inner city areas, benefit from built environments that support physical activity through active transport and recreational opportunities [44]. The proximity of facilities and amenities within walkable distances facilitates regular physical activity, and research demonstrates that improved urban walkability enhances life expectancy while reducing non‐communicable disease burden [45]. In addition, the *healthy migrant effect* may also impact the overall population health of major city where migrants moving to Australia often have to pass strict health checks, are generally younger and healthier and choose to reside in major cities [46].

The elevated obesity rates observed in inner and outer regional areas may be partly explained by employment‐related travel patterns. Residents in these areas often face longer commutes to access employment opportunities, and previous research has shown that commuting distances predict obesity risk. For instance, residents of outer Sydney travelling more than 45 min to work were twice as likely to be obese compared to those with shorter commutes [47]. Extended commuting times likely reduce available time for food preparation, which are predominantly car‐dependent, are associated with diet quality, eating habits and time constraints represent a commonly cited barrier to healthy meal preparation [48]. Aggravating this status quo are likely to be areas replete with fast food outlets offering quick low‐cost high‐calorie and low nutrition meals, with research indicating just a modest ratio of unhealthy to healthy food vendors being associated with higher BMI [49]. Similarly, Australian research indicates that greater supermarket access in urban areas is associated with healthy weight maintenance, though this relationship varies between cities [50]. It is important to note that unhealthy dietary behaviours contribute to increased obesity prevalence across all Australian geographic settings—urban, remote and rural—regardless of food access levels [51], suggesting that availability alone does not determine dietary quality and eating habits. We also note attributing obesity changes to commuting patterns and food environments is plausible but speculative.

### 4.3 Strengths and Limitations

This study presents several notable strengths. We utilised population‐level data with consistent methodological approaches across a decade‐long observation period, enabling robust trend analysis. Median age of death calculations were based on comprehensive mortality records, providing accurate estimates for small geographic areas throughout Australia. Obesity prevalence data were derived from national health surveys using objective measurements and age‐standardised modelling approaches, with consistent modelling approaches over the full study period. The linkage of small‐area datasets allowed for examination of changes by remoteness classification while controlling for area‐level socio‐economic factors.

4.4

Several limitations warrant consideration the obesity data represent small‐area modelled estimates, with larger geographic aggregation units (PHAs) in remote and rural areas due to smaller population sizes. To address this limitation, median age of death data were pooled across multiple years to ensure data reliability and minimise random fluctuations inherent in small‐area statistics. The most recent data used for this study (period 2018–2022) includes 2 years affected by the pandemic; however, this represents the most current data available for comprehensive geographic analysis at this spatial resolution from PHIDU. The use of pre‐aggregated data is a limitation of this analysis as it prevented sensitivity analyses examining alternative temporal groupings or the isolated impact of the COVID‐19 pandemic period. The obesity prevalence data are modelled estimates based on individuals who participated in the ABS National Health Survey rather than direct measurements, which introduces uncertainty particularly in remote areas with smaller populations and limited primary data collection. The precision of these estimates varies geographically, with potentially wider confidence intervals in areas with sparser data. However, the estimates used objectively measured heights and weights, and although measurement uncertainty may affect the magnitude of observed inequities, it is unlikely to alter the overall direction of trends between broad area typologies.

The socio‐economic relative deprivation measure (SEIFA) was calculated for our aggregate spatial unit (PHA) for 1 year only, and we recommend that this measure is computed across the additional SEIFA time‐periods (2011) to allow this measure to align with temporal outcome data. In addition, this is a descriptive study leveraging a repeat cross‐sectional study design and should not be used to infer causal relationships.

## 5 Conclusions

Our results demonstrate increasing inequities in both median age of death and obesity prevalence among Australian major cities, regional and remote/rural areas. The temporal patterns of obesity increase vary markedly by remoteness category, with major cities experiencing the smallest increases compared to inner and outer regional areas. Given obesity's established role as a contributor to premature mortality, these geographic disparities in obesity prevalence may contribute to widening median age of death gaps between urban and rural populations.

Despite public health interventions, obesity rates remain high in major cities and continue rising in regional and remote areas. Without systemic reforms addressing underlying determinants, these geographic disparities in obesity will likely exacerbate inequities in diabetes and related chronic diseases, deepening health divides across communities. Prevention strategies should address the unique challenges faced by non‐urban populations, including built environment limitations, employment‐related travel demands, food access barriers and the ongoing effects of selective out‐migration. A comprehensive approach that considers the complex interplay among geographic, social and economic factors will be essential for reducing these persistent health inequities.

## Author Contributions

Conceptualisation: JRO; Methodology: JRO, TAB, XF; Spatial data analysis: JO; Statistical Analysis: JRO; Writing – Original Draft: JRO; Writing – Review & Editing: All.

## Conflicts of Interest

The authors declare no conflicts of interest.

## Supporting information




**Supplementary Table 1:** Model‐ adjusted median age of death by remoteness and year, males, 2010‐14 to 2018‐22.
**Supplementary Table 2:** Model‐ adjusted median age of death by remoteness and year, females, 2010‐14 to 2018‐22.
**Supplementary Table 3:** Model‐ adjusted obesity prevalence by remoteness and year, males, 2011‐24 to 2022.
**Supplementary Table 4:** Model‐ adjusted obesity prevalence by remoteness and year, females, 2011‐24 to 2022.
**Supplementary Table 5:** Slope Index of Inequality (SII) in median age of death and obesity by remoteness, men and women, 2010‐2022.
**Supplementary Figure 1:** Remoteness categories and Population Health Area boundaries, Australia.

## Data Availability

The data that support the findings of this study are openly available in Public Health Information Development Unit (PHIDU) at https://phidu.torrens.edu.au/.
