# Supplementary material for: Growing Inequities in Median Age of Death and Obesity Prevalence Between Australian Major Cities and Remote Areas: A National, Longitudinal, Spatial Analysis
Source: Public Health Chall. 2026 Jan 29;5(1):e70189. doi: 10.1002/puh2.70189 (PMC12854097; doi:10.1002/puh2.70189)
Supplement: Supplementary file 1 — Supplementary Table 1: Model‐ adjusted median age of death by remoteness and year, males, 2010‐14 to 2018‐22. Supplementary Table 2: Model‐ adjusted median age of death by remoteness and year, females, 2010‐14 to 2018‐22. Supplementary Table 3: Model‐ adjusted obesity prevalence by remoteness and year, males, 2011‐24 to 2022. Supplementary Table 4: Model‐ adjusted obesity prevalence by remoteness and year, females, 2011‐24 to 2022. Supplementary Table 5: Slope Index of Inequality (SII) in median age of death and obesity by remoteness, men and women, 2010‐2022. Supplementary Figure 1: Remoteness categories and Population Health Area boundaries, Australia. [file PUH2-5-e70189-s001.docx]

**Growing inequities in median age of death and obesity prevalence between Australian major cities and remote areas: a national, longitudinal, spatial analysis.**

**Supplementary Material**

***Supplementary Table 1. Model- adjusted median age of death by remoteness and year, males, 2010-14 to 2018-22.***

| **Male Median age of death** | **Coef** | **P value** | **LL 95% CI** | **UL 95% CI** |
| --- | --- | --- | --- | --- |
| **Year** | | | | |
| 2010-14 | ref | | | |
| 2013-17 | 0.32 | 0.02 | 0.04 | 0.60 |
| 2015-19 | 0.53 | <0.001 | 0.25 | 0.81 |
| 2016-20 | 1.07 | <0.001 | 0.79 | 1.35 |
| 2017-21 | 1.21 | <0.001 | 0.93 | 1.49 |
| 2018-22 | 1.42 | <0.001 | 1.14 | 1.71 |
| **Remoteness** | | | | |
| Major Cities | ref | | | |
| Inner Regional | -0.28 | 0.48 | -1.03 | 0.48 |
| Outer Regional | -1.80 | <0.001 | -2.85 | -0.75 |
| Remote | -0.10 | 0.91 | -1.86 | 1.66 |
| Very Remote | -6.04 | <0.001 | -7.96 | -4.12 |
| **Area-level deprivation (SEIFA Decile)** | | | | |
| 1 - most deprived | ref | | | |
| 2 | 1.94 | <0.001 | 0.72 | 3.15 |
| 3 | 2.18 | <0.001 | 0.94 | 3.42 |
| 4 | 2.44 | <0.001 | 1.24 | 3.65 |
| 5 | 1.33 | <0.001 | 0.14 | 2.52 |
| 6 | 1.67 | <0.001 | 0.51 | 2.83 |
| 7 | 1.88 | <0.001 | 0.63 | 3.13 |
| 8 | 2.23 | <0.001 | 0.96 | 3.51 |
| 9 | 3.21 | <0.001 | 1.94 | 4.48 |
| 10 - least deprived | 2.33 | <0.001 | 1.01 | 3.64 |
| **State** | | | | |
| New South Wales | ref | | | |
| Victoria | 0.21 | 0.58 | -0.54 | 0.95 |
| Queensland | -1.07 | 0.01 | -1.85 | -0.29 |
| South Australia | 0.97 | 0.07 | -0.06 | 2.00 |
| Western Australia | -2.61 | <0.001 | -3.59 | -1.63 |
| Tasmania | 0.14 | 0.84 | -1.28 | 1.56 |
| Northern Territory | -11.83 | <0.001 | -14.61 | -9.05 |
| Australian Capital Territory | -2.87 | 0.02 | -5.32 | -0.42 |
| **Model-adjusted estimates** | | | | |
| **year#Remotness** | **Margin** | **p value** | **LL 95% CI** | **UL 95% CI** |
| 2010#Major cities | 76.7 | <0.001 | 76.3 | 77.1 |
| 2010#Inner regional | 76.4 | <0.001 | 75.8 | 77.0 |
| 2010#outer regional | 74.9 | <0.001 | 74.0 | 75.8 |
| 2010#remote | 76.6 | <0.001 | 74.9 | 78.3 |
| 2010#very remote | 70.6 | <0.001 | 68.8 | 72.5 |
| 2018#Major cities | 78.1 | <0.001 | 77.7 | 78.5 |
| 2018#Inner regional | 77.0 | <0.001 | 76.3 | 77.6 |
| 2018#outer regional | 76.0 | <0.001 | 75.0 | 76.9 |
| 2018#remote | 74.3 | <0.001 | 72.6 | 76.0 |
| 2018#very remote | 70.0 | <0.001 | 68.1 | 71.8 |

***Supplementary Table 2. Model- adjusted median age of death by remoteness and year, females, 2010-14 to 2018-22.***

| **Female Median age of death** | **Coef** | **P value** | **LL 95% CI** | **UL 95% CI** |
| --- | --- | --- | --- | --- |
| **Year** | | | | |
| 2010-14 | ref | | | |
| 2013-17 | 0.09 | 0.59 | -0.24 | 0.41 |
| 2015-19 | 0.23 | 0.16 | -0.09 | 0.56 |
| 2016-20 | 0.73 | <0.001 | 0.41 | 1.06 |
| 2017-21 | 0.80 | <0.001 | 0.48 | 1.13 |
| 2018-22 | 0.93 | <0.001 | 0.60 | 1.25 |
| **Remoteness** | | | | |
| Major Cities | ref | | | |
| Inner Regional | 0.07 | 0.85 | -0.67 | 0.82 |
| Outer Regional | -0.83 | 0.11 | -1.86 | 0.20 |
| Remote | 1.06 | 0.23 | -0.69 | 2.82 |
| Very Remote | -6.69 | <0.001 | -8.58 | -4.81 |
| **Area-level deprivation (SEIFA Decile)** | | | | |
| 1 - most deprived | ref | | | |
| 2 | 2.10 | <0.001 | 0.96 | 3.24 |
| 3 | 2.38 | <0.001 | 1.21 | 3.54 |
| 4 | 2.51 | <0.001 | 1.38 | 3.64 |
| 5 | 1.75 | <0.001 | 0.63 | 2.87 |
| 6 | 1.86 | <0.001 | 0.77 | 2.95 |
| 7 | 2.22 | <0.001 | 1.05 | 3.40 |
| 8 | 1.98 | <0.001 | 0.78 | 3.17 |
| 9 | 3.12 | <0.001 | 1.92 | 4.31 |
| 10 - least deprived | 2.92 | <0.001 | 1.68 | 4.15 |
| **State** | | | | |
| New South Wales | ref | | | |
| Victoria | 0.11 | 0.76 | -0.59 | 0.81 |
| Queensland | -1.29 | <0.001 | -2.02 | -0.56 |
| South Australia | 0.93 | 0.06 | -0.04 | 1.90 |
| Western Australia | -3.61 | <0.001 | -4.52 | -2.69 |
| Tasmania | -1.32 | 0.05 | -2.65 | 0.01 |
| Northern Territory | -14.86 | <0.001 | -17.46 | -12.26 |
| Australian Capital Territory | -2.67 | 0.02 | -4.97 | -0.37 |
| **Model-adjusted estimates** | | | | |
| **year#Remotness** | **Margin** | **p value** | **LL 95% CI** | **UL 95% CI** |
| 2010#Major cities | 81.8 | <0.001 | 81.4 | 82.2 |
| 2010#Inner regional | 81.9 | <0.001 | 81.3 | 82.5 |
| 2010#outer regional | 81.0 | <0.001 | 80.1 | 81.9 |
| 2010#remote | 82.9 | <0.001 | 81.2 | 84.6 |
| 2010#very remote | 75.1 | <0.001 | 73.3 | 77.0 |
| 2018#Major cities | 82.7 | <0.001 | 82.3 | 83.2 |
| 2018#Inner regional | 81.8 | <0.001 | 81.2 | 82.4 |
| 2018#outer regional | 81.5 | <0.001 | 80.5 | 82.4 |
| 2018#remote | 80.0 | <0.001 | 78.3 | 81.7 |
| 2018#very remote | 73.0 | <0.001 | 71.2 | 74.8 |

***Supplementary Table 3. Model- adjusted obesity prevalence by remoteness and year, males, 2011-24 to 2022.***

| **Male Obesity** | **Coef** | **P value** | **LL 95% CI** | **UL 95% CI** |
| --- | --- | --- | --- | --- |
| **Year** | | | | |
| 2011–12 | ref | | | |
| 2014-15 | -0.05 | 0.84 | -0.58 | 0.47 |
| 2017-18 | 3.05 | <0.001 | 2.53 | 3.58 |
| 2022 | 2.88 | <0.001 | 2.36 | 3.41 |
| **Remoteness** | | | | |
| Major Cities | ref | | | |
| Inner Regional | -1.40 | <0.001 | -2.18 | -0.63 |
| Outer Regional | -1.30 | 0.02 | -2.38 | -0.23 |
| Remote | 0.59 | 0.53 | -1.23 | 2.41 |
| Very Remote | -0.61 | 0.57 | -2.67 | 1.46 |
| **Area-level deprivation (SEIFA Decile)** | | | | |
| 1 - most deprived | ref | | | |
| 2 | -0.26 | 0.58 | -1.16 | 0.65 |
| 3 | -1.90 | <0.001 | -2.82 | -0.97 |
| 4 | -2.13 | <0.001 | -3.03 | -1.22 |
| 5 | -2.48 | <0.001 | -3.37 | -1.58 |
| 6 | -3.31 | <0.001 | -4.18 | -2.43 |
| 7 | -3.75 | <0.001 | -4.69 | -2.82 |
| 8 | -4.72 | <0.001 | -5.67 | -3.77 |
| 9 | -5.90 | <0.001 | -6.85 | -4.95 |
| 10 - least deprived | -7.49 | <0.001 | -8.47 | -6.51 |
| **State** | | | | |
| New South Wales | ref | | | |
| Victoria | -1.87 | <0.001 | -2.42 | -1.32 |
| Queensland | 1.83 | <0.001 | 1.25 | 2.41 |
| South Australia | 0.46 | 0.24 | -0.31 | 1.23 |
| Western Australia | -1.21 | <0.001 | -1.95 | -0.48 |
| Tasmania | -0.65 | 0.23 | -1.71 | 0.40 |
| Northern Territory | -0.39 | 0.76 | -2.83 | 2.06 |
| Australian Capital Territory | 0.67 | 0.48 | -1.18 | 2.51 |
| **Model-adjusted estimates** | | | | |
| **year#Remotness** | **Margin** | **p value** | **LL 95% CI** | **UL 95% CI** |
| 2011#Major cities | 28.5 | <0.001 | 28.0 | 28.9 |
| 2011#Inner regional | 27.1 | <0.001 | 26.4 | 27.7 |
| 2011#outer regional | 27.2 | <0.001 | 26.2 | 28.1 |
| 2011#remote | 29.1 | <0.001 | 27.3 | 30.8 |
| 2011#very remote | 27.9 | <0.001 | 25.8 | 29.9 |
| 2014#Major cities | 28.4 | <0.001 | 28.0 | 28.8 |
| 2014#Inner regional | 31.8 | <0.001 | 31.2 | 32.5 |
| 2014#outer regional | 31.5 | <0.001 | 30.6 | 32.5 |
| 2014#remote | 29.9 | <0.001 | 28.1 | 31.6 |
| 2014#very remote | 30.4 | <0.001 | 27.7 | 33.1 |
| 2017#Major cities | 31.5 | <0.001 | 31.1 | 31.9 |
| 2017#Inner regional | 36.9 | <0.001 | 36.2 | 37.5 |
| 2017#outer regional | 36.9 | <0.001 | 36.0 | 37.8 |
| 2017#remote | 35.0 | <0.001 | 33.3 | 36.8 |
| 2017#very remote | 37.0 | <0.001 | 32.8 | 41.2 |
| 2022#Major cities | 31.3 | <0.001 | 30.9 | 31.7 |
| 2022#Inner regional | 37.3 | <0.001 | 36.6 | 37.9 |
| 2022#outer regional | 38.1 | <0.001 | 37.2 | 39.1 |
| 2022#remote | 34.4 | <0.001 | 32.6 | 36.2 |
| 2022#very remote | 34.5 | <0.001 | 30.6 | 38.4 |

***Supplementary Table 4. Model- adjusted obesity prevalence by remoteness and year, females, 2011-24 to 2022.***

| **Female Obesity** | **Coef** | **P value** | **LL 95% CI** | **UL 95% CI** |
| --- | --- | --- | --- | --- |
| **Year** | | | | |
| 2011–12 | ref | | | |
| 2014-15 | -1.10 | <0.001 | -1.70 | -0.49 |
| 2017-18 | 0.68 | 0.03 | 0.07 | 1.29 |
| 2022 | 0.61 | 0.05 | 0.00 | 1.22 |
| **Remoteness** | | | | |
| Major Cities | ref | | | |
| Inner Regional | -0.54 | 0.23 | -1.42 | 0.34 |
| Outer Regional | 0.09 | 0.89 | -1.14 | 1.32 |
| Remote | 0.49 | 0.64 | -1.59 | 2.57 |
| Very Remote | -1.73 | 0.15 | -4.09 | 0.63 |
| **Area-level deprivation (SEIFA Decile)** | | | | |
| 1 - most deprived | ref | | | |
| 2 | -0.26 | 0.58 | -1.16 | 0.65 |
| 3 | -1.90 | <0.001 | -2.82 | -0.97 |
| 4 | -2.13 | <0.001 | -3.03 | -1.22 |
| 5 | -2.48 | <0.001 | -3.37 | -1.58 |
| 6 | -3.31 | <0.001 | -4.18 | -2.43 |
| 7 | -3.75 | <0.001 | -4.69 | -2.82 |
| 8 | -4.72 | <0.001 | -5.67 | -3.77 |
| 9 | -5.90 | <0.001 | -6.85 | -4.95 |
| 10 - least deprived | -7.49 | <0.001 | -8.47 | -6.51 |
| **State** | | | | |
| New South Wales | ref | | | |
| Victoria | -0.82 | 0.01 | -1.43 | -0.20 |
| Queensland | 0.42 | 0.21 | -0.23 | 1.06 |
| South Australia | 1.46 | <0.001 | 0.60 | 2.32 |
| Western Australia | -1.21 | <0.001 | -2.03 | -0.39 |
| Tasmania | -0.97 | 0.11 | -2.15 | 0.21 |
| Northern Territory | -1.95 | 0.17 | -4.70 | 0.81 |
| Australian Capital Territory | 1.43 | 0.17 | -0.63 | 3.49 |
| **Model-adjusted estimates** | | | | |
| **year#Remotness** | **Margin** | **p value** | **LL 95% CI** | **UL 95% CI** |
| 2011#Major cities | 28.7 | <0.001 | 28.2 | 29.2 |
| 2011#Inner regional | 28.2 | <0.001 | 27.4 | 28.9 |
| 2011#outer regional | 28.8 | <0.001 | 27.7 | 29.9 |
| 2011#remote | 29.2 | <0.001 | 27.2 | 31.2 |
| 2011#very remote | 27.0 | <0.001 | 24.7 | 29.3 |
| 2014#Major cities | 27.6 | <0.001 | 27.1 | 28.1 |
| 2014#Inner regional | 30.5 | <0.001 | 29.8 | 31.3 |
| 2014#outer regional | 29.9 | <0.001 | 28.8 | 31.0 |
| 2014#remote | 29.3 | <0.001 | 27.3 | 31.3 |
| 2014#very remote | 29.6 | <0.001 | 26.5 | 32.7 |
| 2017#Major cities | 29.4 | <0.001 | 28.9 | 29.8 |
| 2017#Inner regional | 34.5 | <0.001 | 33.7 | 35.2 |
| 2017#outer regional | 34.5 | <0.001 | 33.5 | 35.6 |
| 2017#remote | 32.6 | <0.001 | 30.6 | 34.6 |
| 2017#very remote | 35.7 | <0.001 | 30.9 | 40.5 |
| 2022#Major cities | 29.3 | <0.001 | 28.8 | 29.8 |
| 2022#Inner regional | 35.4 | <0.001 | 34.7 | 36.1 |
| 2022#outer regional | 36.5 | <0.001 | 35.4 | 37.6 |
| 2022#remote | 32.9 | <0.001 | 30.9 | 35.0 |
| 2022#very remote | 33.7 | <0.001 | 29.2 | 38.2 |
|  |  |  |  |  |

***Supplementary Table 5. Slope Index of Inequality (SII) in median age of death and obesity by remoteness, men and women, 2010-2022.***

|  | **SII** | **P value** | **LL 95% CI** | **UL 95% CI** |
| --- | --- | --- | --- | --- |
| **Median Age of Death - Men** | | | | |
| 2010-14 | -2.27 | <0.001 | -3.56 | -0.98 |
| 2013-17 | -2.21 | <0.001 | -3.50 | -0.92 |
| 2015-19 | -2.06 | <0.001 | -3.36 | -0.77 |
| 2016-20 | -3.69 | <0.001 | -4.98 | -2.40 |
| 2017-21 | -3.59 | <0.001 | -4.89 | -2.30 |
| 2018-22 | -3.62 | <0.001 | -4.91 | -2.33 |
| **Median Age of Death - Women** | | | | |
| 2010-14 | -1.28 | 0.05 | -2.55 | 0.00 |
| 2013-17 | -1.17 | 0.07 | -2.45 | 0.10 |
| 2015-19 | -1.01 | 0.12 | -2.28 | 0.27 |
| 2016-20 | -2.55 | <0.001 | -3.83 | -1.28 |
| 2017-21 | -2.56 | <0.001 | -3.83 | -1.29 |
| 2018-22 | -2.51 | <0.001 | -3.78 | -1.24 |
| **Obesity Prevalence - Men** | | | | |
| 2011–12 | 1.81 | <0.001 | 0.59 | 3.02 |
| 2014-15 | 4.59 | <0.001 | 3.36 | 5.81 |
| 2017-18 | 9.05 | <0.001 | 7.82 | 10.28 |
| 2022 | 11.67 | <0.001 | 10.44 | 12.90 |
| **Obesity Prevalence - Women** | | | | |
| 2011–12 | 1.53 | 0.03 | 0.15 | 2.91 |
| 2014-15 | 4.80 | <0.001 | 3.42 | 6.19 |
| 2017-18 | 8.97 | <0.001 | 7.58 | 10.37 |
| 2022 | 12.47 | <0.001 | 11.08 | 13.86 |

***Supplementary Figure 1: Remoteness categories and Population Health Area boundaries, Australia***.


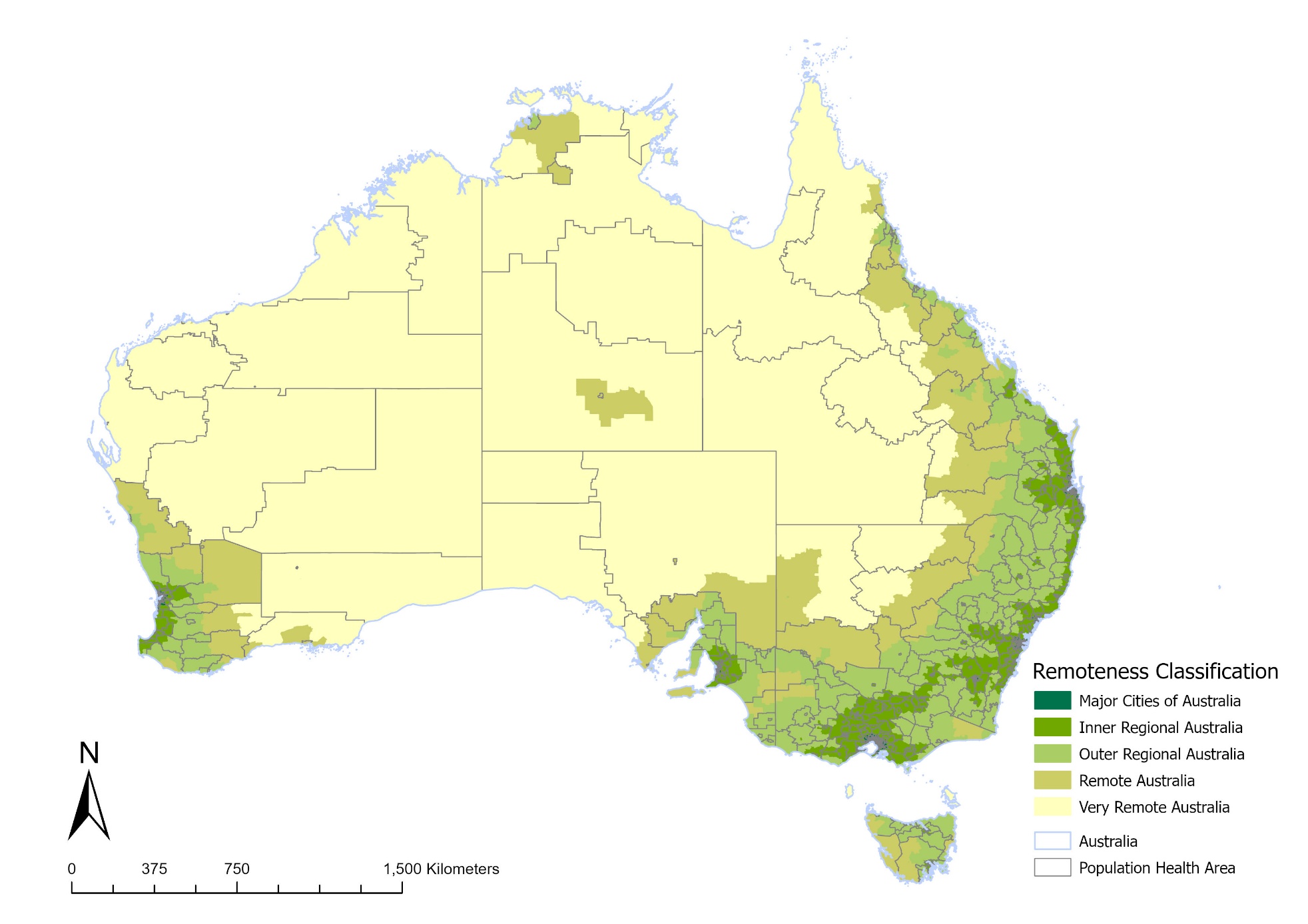


***Supplementary Code and formulas***

**Model 1: Mixed Effects Model**

Formula:

Y_ij = β₀ + β₁(Year_i) + β₂(Remoteness_i) + β₃(Year_i × Remoteness_i)

+ β₄(State_i) + β₅(SEIFA_i) + u_j + ε_ij

Where:

- Y_ij = Median age of death/obesity for observation i in PHA j
- Year_i = categorical time period
- Remoteness_i = categorical remoteness area classification (RACode: 1-5)
- Year_i × Remoteness_i = interaction term testing whether inequalities change over time
- State_i = state/territory fixed effects (ste_code21)
- SEIFA_i = socioeconomic decile fixed effects (seifa_deci)
- u_j ~ N(0, σ²_u) = random intercept for Primary Health Area (PHA)
- ε_ij ~ N(0, σ²_ε) = residual error term

**Stata code:**

**Change over time in median age of death and obesity by remoteness

xtmixed mobese i.year##i.RACode i.ste_code21 i.seifa_deci || phacode:

* Extracttion of marginal means

margins RACode, over(year) atmeans

**Model 2: Slope Index of Inequality (SII)**

Formula:

Y_i = β₀ + β₁(Ridit_i) + β₂(Year_i) + β₃(Ridit_i × Year_i) + β₄(State_i) + ε_i

Where:

- Y_i = Median age of death/obesity for observation i
- Ridit_i = relative position score for remoteness (0.1 for major cities to 0.9 for very remote)
- Year_i = categorical time period
- Ridit_i × Year_i = interaction allowing SII to vary by time period
- State_i = state/territory fixed effects
- ε_i ~ N(0, σ²) = residual error term

**Stata code:**

**Slope Index of Inequality (SII)

xtmixed mle c.ridit##i.year i.ste_code21 i.seifa_deci || phacode:

margins year, dydx(ridit) atmeans
